# The Efficiency of Noni (*Morinda citrifolia* L.) Essential Oil on the Control of Leaf Spot Caused by *Exserohilum turcicum* in Maize Culture

**DOI:** 10.3390/medicines4030060

**Published:** 2017-08-14

**Authors:** Janaina Costa E Silva, Dalmarcia de Sousa Carlos Mourão, Fabia Silva de Oliveira Lima, Renato de Almeida Sarmento, Mateus Sunti Dalcin, Raimundo Wagner de Souza Aguiar, Gil Rodrigues dos Santos

**Affiliations:** 1 Federal Institute of Tocantins, Araguatins Campus, 77950-000 Araguatins, TO, Brazil; janaina.silva@ifto.edu.br; 2 Federal University of Tocantins, Gurupi Campus, 77402-970 Gurupi, TO, Brazil; dalmarciaadm@yahoo.com.br (D.S.C.M.); rsarmento@uft.edu.br (R.A.S.); m2d@uft.edu.br (M.S.D.); rswa@uft.edu.br (R.W.S.A.); 3 Federal Institute of Tocantins, Dianópolis Campus, 77300-000 Dianópolis, TO, Brazil; fabia.lima@ifto.edu.br

**Keywords:** alternative control, medicinal plants, *Zea mays*, *Exserohilum* spot

## Abstract

The objective of this work was to evaluate the efficiency of noni essential oil on the control of *Exserohilum turcicum*, a causative agent of *Exserohilum* spot in maize culture. In the sanitary test 400 seeds were incubated using the blotter test method. For the transmissibility test, the fragments of damaged leaves of seedlings were removed and put into a potato, dextrose and agar (PDA) culture environment. To verify the pathogenicity, Koch´s postulates were performed. In the phytotoxicity test different concentrations of noni oil were applied in maize seedlings. *E. turcicum* conidia were submitted to different concentrations of noni oil. In the preventive and curative tests noni essential oils were applied before and after the conidia inoculation, respectively. The results revealed the presence of fungi of the genres *Aspergillus*, *Penicillium*, *Rhizopus*, *Fusarium*, and *Exserohilum* in the maize seeds. The pathogenicity of *E. turcicum* and also the transmission of this fungus from the seeds to the maize seedlings was confirmed. The inhibition of conidia germination was proportional to the concentration increase. The preventive application of noni essential oil was the most efficient on the control of *Exserohilum* spot.

## 1. Introduction

Maize (*Zea mays* L.) is an annual culture which is very susceptible to the attack of pathogenic fungi transported via seed as it is a species of plant that presents two development phases: one reproductive and other vegetative [1]. Fungi of the *Exserohilum* genre are responsible for causing leaf diseases, and may cause both quantitative and qualitative damage [2]. *Exserohilum turcicum* can cause significant losses because in epidemic seasons it can reach to 100% of the plants [3].

The use of pesticides has been causing serious environmental problems and human health problems, thus encouraging the search for alternative products that could substitute these chemical components [4]. Essential oils have been presented in research as having great potential for phytopathogen control, thus reducing the incidence of pathogenic microorganisms that cause prejudice both in the agriculture and food industries [5]. Essential oils are extracted from plant parts and are formed by a mixture of volatile substances, which are lipophilic and with low molecular weight, and there are many methods for their extraction [6].

Noni (*Morinda citrifolia* L.), belonging to the Rubiaceae family, is a plant native to Malaysia, Australia, India, and southwest of Asia. It is grown in many regions of the world and is well-known to all the people in tropical areas. Ancient Polynesian travelers intentionally carried the noni fruit for treating many health issues that affected the people from that time: wounds, tumors, burns, and menstrual irregularities, among others [7]. While some studies using the extract of this vegetable and evaluating its fungitoxic effect in plants have already been published, research proving the potential of the noni essential oil on the alternative control of diseases in vegetables is still very scarce. As such, the objective of the present work was to evaluate the efficiency of the noni essential oil on the control of the *Exserohilum* leaf spot in maize culture.

## 2. Material and Methods

The experiment was conducted at the Phytopathology Laboratory of Tocantins Federal University, University Campus of Gurupi using maize seeds (creole type) obtained from the lots of five producers present at trade fairs and produced in the year 2015 in the municipality of Gurupi, in the state of Tocantins, Brazil. These seeds were mixed and later utilized in sanitary and transmissibility tests. For the pathogenicity and phytotoxicity preventive and curative tests, maize seeds (hybrid 30F53YH) were treated with fungicide.

Noni ripe fruits collected in the region of Gurupi were washed in running water, cut in small cubes and submitted to the extraction of essential oil by the hydrodistillation method. In a round-bottom flask 200 g of noni ripe fruits were added. Following this, the flask was attached to Clevenger distiller for a two-hour period. After the extraction, the essential oil was collected in the supernatant form, placed in amber bottle, identified, and stored at 4 °C.

### 2.1. Sanitary of the Maize Seeds

After mixing the acquired seeds, these were incubated in a total of 400 seeds by the blotter test method. A completely randomized experimental design was used with eight repetitions. Sixteen previously disinfected gerbox-type acrylic boxes were covered with sterilized filter paper and dampened with sterile water. For each repetition there were two gerbox boxes considered. All the material was conditioned in an incubating chamber under a temperature of 25 °C for seven days, with photoperiod of 12 h of light.

The analyses were performed using a stereoscopic and optical microscope, as well as a specialized manual for identification of imperfect fungi in [8], with data being presented in percentages.

### 2.2. Seed-Seedling Transmissibility of Exserohilum turcicum

A completely randomized experimental design with four repetitions was used. The substrate was constituted by a mixture of washed thin sand and red-yellow latosoil in a proportion of 1:1 that was submitted to disinfection by solarization for seven days. Following this, a total of 400 seeds was sown in eight trays containing the substrate. The humidity was kept by daily irrigation. After a period of 30 days, a germination count was performed in each tray and samples of leaves with spots were removed. After asepsis with alcohol (70%), hypochlorite (1%), and distilled/sterilized water the leaves fragments were incubated through potato, dextrose and agar (PDA) culture. The plates were sealed, identified and taken to incubation chamber at 25 °C with a photoperiod of one hour of light for 10 days.

### 2.3. Pathogenicity of Exserohilum turcicum to Maize Seedlings

The fungus *E. turcicum* was obtained from maize seeds incubated by the blotter test method and transferred to the PDA environment in Petri plates (90 mm) that remained conditioned in incubating chamber for 15 days, with mean temperature of 25 °C and photoperiod of 12 h of light until the mycelial growth reached the plates borders. Subsequently, the conidia originating in the plates were quantified in Neubauer chamber and suspensions of 1.25 × 10^6^ conidia mL^−1^ of *E. turcicum* were obtained. Afterwards, with the aid of a manual sprayer, the suspensions were applied in the leaf limbs of maize seedlings. The inoculated plants were conditioned in a humid, dark chamber, proportioned by a plastic bag, with cotton soaked in sterile water for a period of 48 h at 25 °C, and later were kept in ambient temperature for seven days.

Aiming at complying with Koch´s postulates, the damaged fragments that appeared on the leaves after four days of the inoculation were removed. Afterwards, asepsis of the damaged fragments in alcohol (70%), hypochlorite (1%) and sterile water was performed. Later, plating was performed in the PDA environment. The plates were sealed with paper film, identified, and taken to incubation chamber for a period of seven days for verification of the phytopathogen growth. 

### 2.4. Chromatographic Analysis of the Noni Essential Oil

The chromatographic analysis was realized at the Federal University of Viçosa, in the state of Minas Gerais, Brazil. The qualitative and quantitative analyses of the essential oils were performed using chromatography in gas phase attached to the mass spectrometry GC-M.S. The chromatograph utilized was the model Shimadzu GC-210 equipped with mass selective detector model QP2010 Plus, and was operated in the following conditions: a capillary column of fused silica TRX-5MS (30 m × 0.25 mm × 0.25 μm of film thickness), with the following programming of the temperature on the column: 60–240 °C (3 °C min^−1^), injector temperature: 220 °C; carrier gas helium, and splitless injection with injected volume of 1 μL of a solution 1:1000 in hexane. For the mass spectrometer (EM), the following conditions were utilized: impact energy of 70 eV, and ion source and interface temperature: 200 °C. In the sample conditions, a homologous series of n-alkanes (C9H20 – C26H54) was injected. The obtained specters were compared with the Nist and Wiley 229 Library’s database and the retention rate calculated for each component was compared to that tabulated, according to [9]. The quantification of the component levels was expressed in a percentage assuming base area normalization, which was obtained with a gas chromatograph equipped with a flame ionization detector, using a Shimadzu GC-210 device, in the following conditions. Capillary column RTX-5MS (30 m × 0.25 mm × 0.25 μm of film thickness); injector temperature: 220 °C; DIC temperature: 300 °C. Column programming: initial temperature of 60 °C with a heating rate of 3 °C min^−1^ until 240 °C, next passing to a heating rate of 10 °C min^−1^ until 300 °C and remaining at this temperature for 10 min; drag gas nitrogen (1.18 mL min^−1^); split rate 1:50; column pressure of 115 KPa and injected volume of 1 μL in hexane.

### 2.5. Phytoxicity in Maize Seedlings Using Different Concentrations of Noni Essential Oil

Maize seeds of the hybrid 30F53YH treated with fungicide were sown in polyethylene vases constituting four repetitions. A completely randomized experimental design was used with eight treatments (concentrations of noni essential oil of 0.0%, 0.1%, 0.25%, 0.5%, 0.75%, 1%, 1.25%, and 1.5%) and three repetitions. The 10-mL solution from each concentration of noni essential oil (diluted in Tween 80 to 1%) was applied to the seedling leaves with the aid of a sprayer, and the analysis of the phytotoxic effect was realized 48 h after its application.

For the evaluation of phytotoxicity the adapted scale from [10,11,12] was used, with 0 = for absence of phytotoxicity; 1–25 = light chlorosis or beginning of necrosis on the seedlings leaves; 26–50 = medium chlorosis or necrosis on the seedlings leaves; 51–75 = high chlorosis or necrosis on the seedlings leaves; and 76–100 = wilt and dryness of the plant.

### 2.6. Conidia Germination of Exserohilum turcicum According to Different Concentrations of Noni Essential Oil

The utilized design was completely randomized experimental with five treatments (concentrations of noni oil in the order 0.0%, 0.1%, 0.25%, 0.5%, and 0.75%) and four repetitions. For each treatment one mixture was used, in a sterilized flask of 1000 µL of conidia suspension and 1000 µL of the solution of noni essential oil. Just after, the flasks were conditioned in an incubating chamber which remained for a period of 96 h (four days) for the evaluation of the germination. After this period, the conidia solutions were pipetted and put in a Neubauer chamber constituted by four quadrants, where each quadrant constituted one repetition. For conidia germination counting, 100 total conidia (germinated and non-germinated) were counted in the view field of the lens at 10× of the optical microscope, in different positions for each quadrant. The percentage of conidia germination was calculated. The data was not appropriate to any model for the regression equations, so the original data were used.

### 2.7. Preventive and Curative Control of Exserohilum turcicum Spot According to Different Concentrations of Noni Essential Oil

The design used was completely randomized experimental with five treatments (concentrations of noni oil in order: 0, 0.1, 0.25, 0.5, and 0.75%), four repetitions, and five evaluation seasons.

Maize seeds of the hybrid 30F53YH in plastic vases were sowed and, after 45 days, there was an application of the treatments. The concentrations were obtained by the mixture of noni essential oil and water with Tween 80 (1%). For the attainment of the conidia suspension, 10 mL of water was put in each plate in a total of 12 Petri plates and with the aid of a soft brush mycelium detachment was performed. The solution was filtered with gauze and the conidia were quantified in the Neubauer chamber. The data were adjusted thus obtaining a mean of 1.86 × 10^6^ conidia/mL.

In the preventive control test as a spray the five treatments with the noni oil concentrations were initially applied. After two hours on the leaves a solution of 10 mL of the conidia of *E. turcicum* was pulverized on each maize seedling. Following, the vases were submitted to the humid and dark chamber at 25 °C for 48 h. For the curative control, initially in the leaves of maize seedlings 10 mL of the solution containing the fungus conidia were applied. Subsequently, the seedlings were also submitted to the humid and dark chamber for a period of 48 h and after this period treatments with the oil concentrations were applied. All the treated plants, both the preventive control and the curative, were exposed to a natural environment (temperature 30 °C ± 4 °C mm, air relative humidity between 25 and 35%) for a period of ten days, with evaluations being done in intercalated periods of 48 h.

The evaluations of severity of the disease were realized with the note scale adopted by [13] where 0 = healthy plant; 1 = less than 1% of leaf area sick; 3 = 1 to 5% of the leaf area sick; 5 = 6 to 25% of leaf area sick; 7 = 26 to 50% of leaf area sick; 9 = more than 50% of leaf area sick. The severity notes were utilized for attainment of the Area Under Disease Progress Curve (AUDPC), according to [14]. With the AUDPC determined, it was plotted versus the concentration in order to represent the disease evolution according to the noni essential oil concentrations on the preventive and curative control. The data were submitted to regression calculations for the coefficient of determination and the model that was better adjusted to the data was from the quadratic equation. 

## 3. Results

### 3.1. Sanity of Maize Seeds

**Figure 1:**
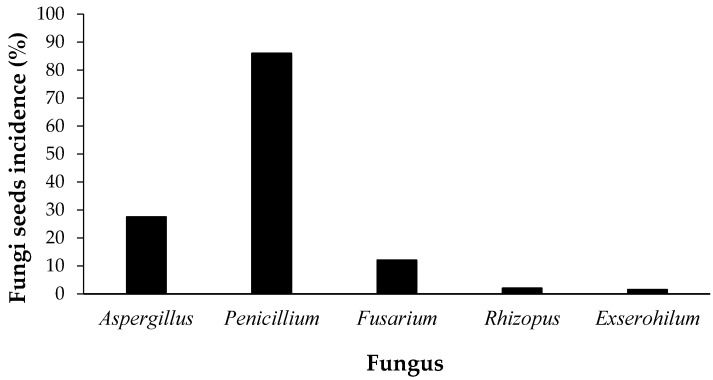
Sanity of maize (*Zea mays* L.) seeds obtained from the trade fair in the municipality of Gurupi-Tocantins, in the year 2015.

The analyses revealed that the fungi transported by seeds were *Aspergillus* sp., *Penicillium* sp., *Fusarium* sp., *Rhizopus* sp., and *E. turcicum*, with respective incidences of 27.5, 86, 12, 2, and 1.5% (Figure 1). The genres *Fusarium* and *Exserohilum* are considered phytopathogenics, and the others are contaminants or present in stored grains.

### 3.2. Seed-Seedling Transmissibility of Exserohilum turcicum

The transmission of *E. turcicum* from the seeds to the seedlings was confirmed with incidence on this pathogen in 4% of the maize seedlings. These presented symptoms typical of the disease caused by this pathogen, such as initially elliptical, straw-colored damaged areas with well-defined borders. Afterwards, with the evolution of the disease the damaged areas became dark with a long format. The plates of culture environment with fragments of the damaged leaves proved the presence of the pathogen, and the mycelium grew in all the samples showing through this test its capacity for being transmitted via seed, causing leaf damage typical of the *Exserohilum* spot.

### 3.3. Pathogenicity of Exserohilum turcicum to Maize Seedlings

The fungus *E. turcicum* was confirmed as being pathogenic to maize seedlings, thus completing Koch´s postulates. The leaves where inoculated the fungus conidia presented initially dark-brown necrotic elliptical spots, which later evolved to big damages with long format and with the aspect of dry leaves characteristic of the disease.

### 3.4. Chromatographic Analysis of Noni Essential Oil

The components of the noni essential oil were identified comparing its mass specters with those from databanks of the Nist and Wiley 229 libraries. Some components had their identities confirmed by comparison between the retention rates calculated with those present in webbook [15] and with the literature [9]. Table 1 shows the relative percentage (area %) of the components of noni essential oil and indicates that the major component found was octanoic (or caprylic) acid (82.24%), followed by hexanoic (or caproic) acid (8.26%).

### 3.5. Phytotoxicity in Maize Seeds Using Different Concentrations of Noni Essential Oil

For the phytotoxicity test using dosages of 0.1 to 1.5% of noni essential oil, it was observed that symptoms of phytotoxicity only occurred in seedlings where the concentrations 1, 1.25, and 1.5% were applied (Table 2). For the three concentrations, the seedlings presented numerous leaves with chlorosis and necrosis. The leaf sheath and veins regions, where the oil accumulated, presented much more evident symptoms. 

The seedlings where were applied the concentrations 0.1, 0.25, 0.5, and 0.75% of the oil did not present symptoms of phytotoxicity. The control only with water and Tween 8 (1%) also did not present symptoms, demonstrating thus that the disperser did not cause any kind of damage to the plant tissue.

### 3.6. Effect of Noni Essential Oil in Exserohilum turcicum Conidia Germination

**Figure 2:**
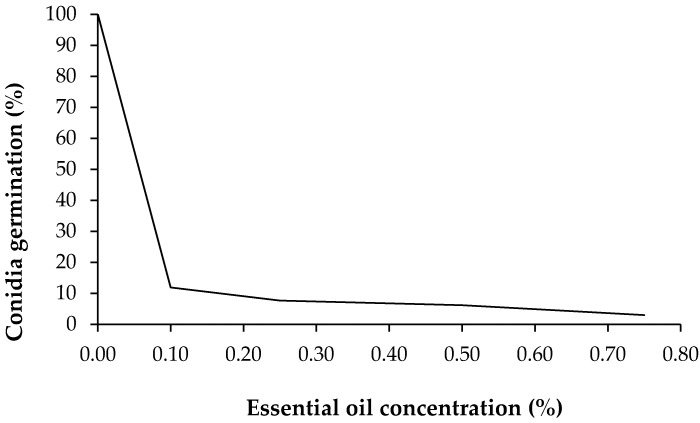
*Exserohilum turcicum* conidia germination in order of rising concentrations of noni (*Morinda citrifolia* L.) essential oil.

The inhibition of conidia germination was proportional to the increase of concentrations (Figure 2). Considering the control with 100% of conidia germination after four days of incubation and comparing it with the germination of conidia submitted to the treatments, the test revealed that at 0.1% of oil concentration there was germination of 11.91%; at 0.25% germination of 7.66% occurred; at 0.5% concentration there was a germination of 6.18%, and the higher concentrations of noni essential oil, 0.75%, presented higher inhibition, with only 2.98% of germination for this concentration. The phytopathogens, when sending out the germination tubes in the vegetable tissues, have the capacity to produce enzymes that cause serious alterations in metabolism, which can lead to plant death.

### 3.7. Preventive and Curative Control of the Exserohilum Spot in Order of Different Concentrations of Noni Essential Oil

**Figure 3:**
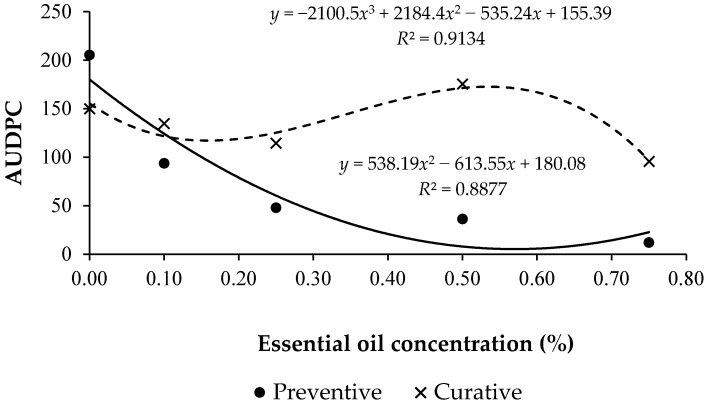
Area under disease progress curve (AUDPC) for the preventive and curative control of *Exserohilum* spot in order of different concentrations of noni (*Morinda citrifolia* L.) essential oil. Gurupi-Tocantins, 2015.

The evaluations of severity of the disease showed that the preventive effect was positive in the control of *Exserohilum* spot, which did not occur in relation to the curative effect (Figure 3).

In the preventive test, the severity of the disease was proportional to the increase of concentrations of noni essential oil. Thus, the concentration 0.75% was the best at controlling the fungus action, and the 0.1% concentration, which among the four concentrations of oil was the one that had higher severity, presented an AUDPC value inferior to the half of the control value. All the concentrations presented values significantly lower than those presented by the control.

In the curative test, the concentrations of 0.1, 0.25, and 0.75% presented AUDPC values lower than for the control, a lower efficiency when compared to the preventive effect. However, the concentration of 0.5% presented, on the severity scale, values higher than for the control. Thus, it is not possible to affirm that the noni essential oil had a positive effect in the curative control of the disease, since one of the highest concentrations applied was not efficient, and the others did not demonstrate values so different to those presented by the control.

## 4. Discussion

In all seeds pathogenic or non-pathogenic fungi were detected. The transport and transmission of these fungi by the seed is a mechanism of survival and dissemination of these pathogens to new areas [16]. When analyzing the sanitary quality of maize seeds, the presence of the fungi *Fusarium* sp., *Cladosporium* sp., *Aspergillus* sp., *Cephalosporium* sp., and *Penicillium* sp was verified. Research shows that the fungi of the genres *Fusarium*, *Aspergillus* and *Penicillium* are the most common in maize seeds. Other authors [17] comment that the pathogen *E. turcicum* survives from one year to another in culture remains (in mycelium and conidia forms), seeds, reminiscent plants, or in alternative carriers.

According to the authors of [18] the fungi associated to seeds may be transported by infestation or infection. The infestation is caused by contaminant fungi which adhere to the seed surfaces. However, the infection is caused by fungi that are found internally in the seed tissue, being the causative agents of diseases in plants.

Casela and collaborators [19] affirm that the typical symptoms of diseases caused by the *Exserohilum* genre occur initially in the inferior leaves, with necrosis of coloration varying from grey-green to brown. When inoculating conidia from ten isolates of *E. turcicum* in maize seeds, the authors of [20] verified, after 15 days, a mean of 2.5 damaged areas/leaf; of 39.7 × 3.4 mm length and width respectively and 4% severity, thus proving that all of them were pathogenic to the culture. The disease *Exserohilum* spot occurs in regions of moderate climates (18 to 27 °C) such as in the south region and in the plateaus of the center–west region, since they have a favorable climate for its growth [21]. It was observed that the disease has been affecting the maize culture in the south of the state of Tocantins, although it is a region of typically hot climate. The possible cause of the increase in the severity in diseases in the state of Tocantins is a favorable climate [22]. These authors also point out that the identification of leaf diseases, the understanding of the aspects related to its development, and the knowledge of efficiency of the control actions are fundamental for the expansion of maize culture in the state.

Sarmento-Brum and collaborators [4], when evaluating the phytotoxic effect of lemongrass and citronella essential oil on watermelon (*Citrullus lanatus*) plants, noted that the concentration of 1% presented low phytotoxicity, and the concentrations of 2 and 4% were highly phytotoxic with the appearance of necrosis on the leaves, mainly in the regions in which the solution of essential oil were accumulated, like the borders and in leaf veins. Furthermore, the authors point out that the secondary metabolites, besides presenting great potential in the use of sustainable agriculture, may be prejudicial in intervening the process of development of the plant.

The necrosis derived from the phytotoxic action of essential oil reduce the leaf area intervening the photosynthetic process and, consequently, preventing the production of substances that are essential energy sources for vegetable survival. The authors of [23] point out that secondary metabolites may bring serious prejudices that disturb vegetable development. They may cause rupture of the membranes, reducing the organelles, preventing the synthesis of chlorophyll and significantly affecting the photosynthetic process.

As previously shown, the major component of the noni essential oil was octanoic acid. This component, in higher concentrations, may have effected an inhibiting action upon the chlorophyll pigment, provoking initially aspects of yellowness along with the cell membrane ruptures, thus causing the wilting of the leaves.

The cinnamon (*Cinnamomum verum*) and citronella (*Cymbopogon winterianus*) essential oils reduced the germination of *Cercospora coffeicola* 16 h after the inoculation, fostering, in some cases, the overflow of cell content observed in electronic microscopy [24]. Other authors [25], based on experiments, concluded that the red guava (*Psidium guajava*), lippia (*Lippia alba*), lemongrass (*Cymbopogon citratus*), white guava (*Psidium guajava*), wide-leaf basil (*Ocimum basilicum*), clove (*Syzygium aromaticum*), pepper-rosemary (*Lippia sidoides*), and wild rosemary (*Eriocephalus africanus*) extracts caused a 100% inhibition of the germination of *Colletotrichum gloeosporioides*, a causal agent of anthracnose in the passionfruit tree. The authors of [26] noted that the extracts of yarrow (*Achillea millefolium*), lemongrass (*Cymbopogon citratus*), camphor (*Cinnamomum camphora*), and rosemary (*Rosmarinus officinalis*) had a fungitoxic effect upon the growth of the *E. turcicum* germination tube.

The results have shown that the major component, octanoic acid, may have been responsible for the effect of inhibition of *E. turcicum* germination, in a way that the higher oil concentration had a more significant effect.

Lima [27] verified, in his works, that citronella essential oil had its effect limited in the concentrations of 2000 ppm, thus demonstrating that the oil did not have an effect upon the fungus action when it was already in the interior of the plant tissue. For the preventive effect, when analyzing AUDPC, the author noted that citronella oil was efficient in the control of cotton ramulosis. In another study [28] the authors emphasize that use of citronella (in terms of curative effect) at a concentration of 2% resulted in decrease of symptoms of the disease in 50% of the plants. In terms of the preventive effect, these authors verified that in citronella essential oil concentrations of 1.5, 1.75, and 2% applied in rice plants, there were no symptoms of the disease found.

The highest values of the oil concentration provided lower values for AUDPC for the preventive control and showed better adjustment of the determination coefficient. The curve evidenced a lower severity in the concentration of 0.75%. In the curative effect the AUDPC was found to be elevated, and there was no value-decreasing scale in relation to the increase of the concentrations of essential oil found in the preventive control.

## 5. Conclusions

It was concluded with this work that in the sanity test of maize seeds the fungi *Penicillium* sp., *Aspergillus* sp., *Rhizopus* sp., *Fusarium* sp., and *E. turcicum* were found, with the latter transmitted via seed-seedlings. *E. turcicum* was pathogenic to the maize seedlings. The major component of noni essential oil was octanoic acid. Concentrations above 1% of the noni essential oil were phytotoxic to the maize seedlings. The concentrations of the noni essential oils were efficient in the inhibition of the *E. turcicum* germination. The preventive application of the noni essential oil was more efficient in the control of *Exserohilum* spot than the curative application.

## Figures and Tables

**Table 1:** Relative percentage (area %), obtained by gas chromatography attached to a mass spectrometry detector, of the components of ripe noni (*Morinda citrifolia* L.) fruit essential oil.

Composts	CN	RT	RI	(%)
3-Methyl-3-butenyl-1-acetate	1	4.583	888	- *
2-Heptanone	2	4.992	897	-
Methyl hexanoate	3	5.774	922	-
Hexanoic acid	4	7.634	987	8.26
Ethyl hetanoate	5	7.974	999	2.48
Methyl octanoate	6	12.713	1123	-
Octanoic acid	7	15.603	1177	82.24
Ethyl octanoate	8	15.803	1196	-
Isopentyl hexanoate	9	18.537	1259	1.6
3-Methyl-2-butenyl hexanoate	10	19.983	1292	-
Not identified	11	24.026	-	-
3-Methylbutyl octanoate	12	26.897	1457	4.25
3-Methylbutyl-2-enyl octanoate	13	28.226	1489	-
Essential oil content (%)				0.20

CN = Composts number; RT = Retention temperature; RI = Retention index; * Not quantified (values < 0.05).

**Table 2:** Phytotoxicity in maize seeds in order to the application of different concentrations of the noni essential oil. Gurupi-Tocantins, 2015.

Treatments	Caracteristics
0.00%	Phytotoxicity absence *
0.10%	Phytotoxicity absence
0.25%	Phytotoxicity absence
0.50%	Phytotoxicity absence
0.75%	Phytotoxicity absence
1.00%	51–75% High chlorosis or high necrosis on the plants stem
1.25%	51–75% High chlorosis or high necrosis on the plants stem
1.50%	51–75% High chlorosis or high necrosis on the plants stem

* Adapted scale of [10,11,12].
